# *Ras^V12^; scrib^−/−^* Tumors: A Cooperative Oncogenesis Model Fueled by Tumor/Host Interactions

**DOI:** 10.3390/ijms22168873

**Published:** 2021-08-18

**Authors:** Caroline Dillard, José Gerardo Teles Reis, Tor Erik Rusten

**Affiliations:** 1 Centre for Cancer Cell Reprogramming, Faculty of Medicine, Institute of Clinical Medicine, University of Oslo, 0372 Oslo, Norway; j.g.b.t.r.d.reis@medisin.uio.no; 2 Department of Molecular Cell Biology, Institute for Cancer Research, Oslo University Hospital, Montebello, 0379 Oslo, Norway

**Keywords:** cold-blooded cancer, cooperative oncogenesis, tumor/stroma interactions

## Abstract

The phenomenon of how oncogenes and tumor-suppressor mutations can synergize to promote tumor fitness and cancer progression can be studied in relatively simple animal model systems such as *Drosophila melanogaster.* Almost two decades after the landmark discovery of cooperative oncogenesis between oncogenic *Ras^V12^* and the loss of the tumor suppressor *scribble* in flies, this and other tumor models have provided new concepts and findings in cancer biology that has remarkable parallels and relevance to human cancer. Here we review findings using the *Ras^V12^; scrib^−/−^* tumor model and how it has contributed to our understanding of how these initial simple genetic insults cooperate within the tumor cell to set in motion the malignant transformation program leading to tumor growth through cell growth, cell survival and proliferation, dismantling of cell–cell interactions, degradation of basement membrane and spreading to other organs. Recent findings have demonstrated that cooperativity goes beyond cell intrinsic mechanisms as the tumor interacts with the immediate cells of the microenvironment, the immune system and systemic organs to eventually facilitate malignant progression.

## 1. Introduction

Mutations in oncogenes and tumor suppressors can synergize over time in order to optimize the tumor fitness within the host, a phenomenon known as cooperative oncogenesis. For example, cooperation between Kras and Myc has been shown to enhance tumorigenesis and weaken immunosurveillance [1]. Pharmacological inhibition of the cooperation between Kras and mutated p53 has proven efficient in decreasing metastasis [2]. Although these findings highlight the importance of genetic cooperation for malignant transformation, the precise mechanisms operating during cooperative oncogenesis remain unclear.

To investigate unconventional synergizing networks and their physiological outcomes, *in vivo* models are essential. Because of its short life cycle, the tremendous collection of genetic tools available, and the remarkable conservation of most signaling pathways, *Drosophila* is a relevant model for studying oncogenesis. During the last decades, cancer research using *Drosophila* has dramatically increased [3]. *Drosophila* cancer models as diverse as gut, brain, hematopoietic, and carcinoma models have been developed and exhibit typical hallmarks of cancer [4]. Although some aspects of cancer biology are difficult to model in flies, such as angiogenesis or immunosurveillance, genetic and chemical screens using *Drosophila* have brought new insights on fundamental cancer biology and the identification of therapeutic targets [3,5].

**Figure 1:**
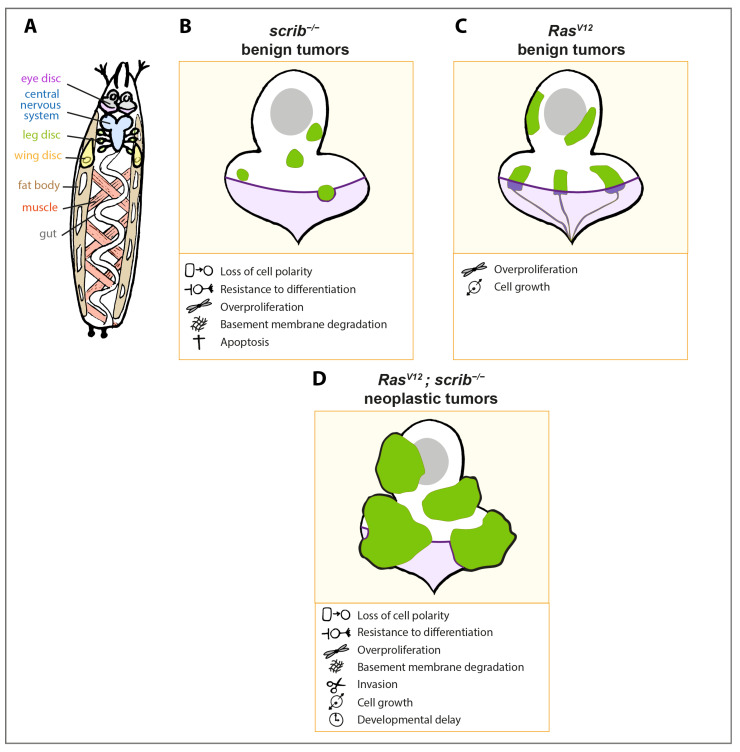
The cooperation between the oncogene *Ras^V12^* and loss of the tumor suppressor *scribble* drives the malignant transformation of eye disc epithelial cells. (**A**) Representation of a larva with most of its organs. An eye-antennal disc is composed of two main parts. The anterior part is the antennal part (white and grey), which will transform into the antenna and part of the head during metamorphosis. The posterior part is the eye part which generates the photoneurons (purple) in a synchronous manner after the morphogenetic furrow (purple line). (**B**) *scrib^−/−^* clones (green) form benign tumors characterized by a loss of cell polarity, resistance to differentiation into neurons (accompanied by a distortion of the morphogenetic furrow), overproliferation, and basement membrane degradation. Ultimately, they are eliminated through apoptosis. (**C**) *Ras^V12^* clones (green) form hyperplastic tumors (defined as tumors that overgrow without disrupting the tissue architecture and differentiation). They are characterized by overproliferation and cell growth. Because neuronal differentiation is not impaired within *Ras^V12^* clones, transformed cells differentiate synchronously to their *wt* neighbors (purple cells within the clones) at the morphogenetic furrow (thick purple line) and form long neuronal axons (purple lines) that extend through the posterior part of the eye disc in order to form the optic nerve. (**D**) *Ras^V12^; scrib^−/−^* clones form neoplastic tumors (defined as tumors that disrupt the tissue morphology and impair differentiation). They are characterized by a loss of cell polarity, resistance to differentiation, overproliferation, cell growth, basement membrane degradation, and invasion. *Ras^V12^; scrib^−/−^* tumors trigger a developmental delay, postponing pupation.

Imaginal discs from the *Drosophila* larva constitute a site of choice for investigating the biology of carcinoma—the most common cancer type, which originates from epithelial cells (Figure 1A).

Imaginal discs are simple epithelial layers set aside during embryogenesis. They undergo controlled growth, patterning, differentiation, and morphogenesis during larval and pupal stages to make specific adult organ structures. Due to evolution, the molecular language that controls these processes is conserved among metazoa, from fly to man. It is the regulated growth, patterning, differentiation, programmed cell death, and tissue structure that fall apart during tumorigenesis. This deconstruction can be studied in flies. More importantly, a vast number of studies in the wing and eye-antennal discs have revealed several sets of cooperating genes that control tumor formation, growth and survival (reviewed in [6]). 

Among these cancer models, the cooperation between the *Drosophila* KRAS homolog oncogene, *Ras^V12^* and the loss of the cell polarity tumor suppressor *scribble* (referred to hereafter as “*Ras^V12^; scrib^−/−^*”) has been the most extensively investigated. This model recapitulates essential aspects of carcinoma development: loss of epithelial structure and differentiation, sustained proliferation, cytoskeletal remodeling, resistance to cell death, basement membrane degradation, and invasion [7,8,9,10,11]. The study of this specific cooperation has been particularly fruitful, providing novel concepts and mechanisms that go beyond intracellular cooperation.

In this review, we summarize the contribution of the *Ras^V12^; scrib^−/−^* model to the understanding of cooperative oncogenesis and cancer biology. We start with the first observations of the cooperation between *Ras^V12^* oncogene expression and the loss of cell polarity. We then review how intrinsic cues contribute to the growth of malignant *Ras^V12^; scrib^−/−^* tumors. Subsequently, we highlight the importance of local interactions between the tumor and its microenvironment for regulating its fate and progression. Finally, we consider the effect of long-range tumor–host interactions for sustaining tumor growth and transformation.

## 2. Establishment of the *Ras^V12^; scrib^−/−^* Cooperative Model

The *Ras^V12^; scrib^−/−^* tumor model is a good illustration of the concept of cooperative oncogenesis. Alone, each insult (loss of cell polarity or *Ras^V12^* overexpression) gives rise to slow-growing non-invasive benign tumors. However, together, they trigger the growth of massive invasive malignant tumors that ultimately kill the host. In this part, we describe the first observations that led to the establishment of this cooperative oncogenic model and their relevance to mammalian cancers. 

### 2.1. Loss of the Cell Polarity Gene Scribble Triggers the Formation of Benign Tumors

Downregulation and mislocalization of cell polarity proteins is typically observed in many human tumors (reviewed in [12]). It often appears during the early stages of malignancy [13,14] and is associated with aggressive cancers [15,16,17,18,19,20,21]. Conversely, polarity gene amplification has been reported in several human cancers. These opposite observations highlight the need for a better understanding of the function of cell polarity regulators in cancer and the characterization of their downstream effectors in different contexts [22].

Studies in *Drosophila* have allowed the discovery of the Scribble polarity tricomplex, which is composed of the LAP scaffolding protein Scribble (Scrib), the MAGUK protein Disc large (Dlg), and the WD-repeat protein Lethal giant larvae (Lgl). In *Drosophila* and mammals, the three proteins are located at the basolateral side of epithelial cells (Reviewed in [23]). Although physical interactions between these proteins are not yet fully understood, genetic interactions demonstrated that they function together to establish and maintain apicobasal polarity [24]. Apicobasal polarity is essential to define specific membrane compartments which restrict local signal transduction. In this way, individual epithelial cells can integrate cues from their neighboring cells within the epithelium and adjust their proliferation, growth, survival, metabolism, and motility to ensure proper tissue homeostasis (reviewed in [12]). The human orthologs of Scrib, Dlg, and Lgl play similar roles in mammals, and they can substitute for their fly counterparts and rescue loss of cell polarity defects in *Drosophila* [25,26,27,28,29]. However, their precise modes of action seem more complex, probably due to both redundancies between paralogs and tissue specificities (reviewed in [30]). Interestingly, upon infection, the Human Papilloma Virus targets Scrib and Dlg for degradation, stressing their potential role in tumor formation [31]. 

Tumor formation can be modeled in *Drosophila* eye discs using the MARCM technique (Mosaic Analysis with a Repressible Cell Marker) [32]. With this genetic trick, randomly generated individual cells undergo loss of heterozygosity upon mitotic recombination. As a consequence, they can inherit two identical copies of a loss-of-function allele for a tumor suppressor. At the same time, the MARCM technique allows to label the cells with GFP and optionally to drive the expression of any gene of choice, such as an oncogene. Thus, both the loss of a tumor suppressor and the gain of function of an oncogene can be modeled from a single tumor-initiating cell, mimicking the early random genetic events that initiate tumorigenesis in mammals.

MARCM *scrib^−/−^* cells form small tumors within the eye disc (Figure 1B). They are characterized by a loss of cell polarity, cell morphology alterations (small round cells, multilayered clones, downregulation of DE-Cadherin), overproliferation involving CyclinE upregulation, and resistance to differentiation (Brumby and Richardson, 2003). These clones, nevertheless, remain small due to their active elimination through apoptosis. As a result, the adult flies harbor a smaller but functional eye. Interestingly, cell polarity mutant clones also display some preinvasive traits. However, despite enrichment for the Matrix Metalloproteinase 1 (MMP1) and subsequent basement membrane degradation, *scrib^−/−^* cells are unable to invade other tissues [7,10,11,33,34]. 

These observations demonstrate that the loss of *scrib* is not sufficient to drive malignancy in the *Drosophila* imaginal discs on its own.

### 2.2. The Expression of the Oncogene Ras^V12^ Alone Is Not Sufficient to Create Neoplastic, Invasive Tumors

Ras is a membrane-associated guanine nucleotide-binding protein. Upon activation by different Receptor Tyrosine Kinase (RTK) growth factor receptors, Ras changes its conformation from a GDP-bound inactive form to an active GTP-bound form that relays the signal to diverse downstream effectors; the MAPK pathway, the PI3K-AKT-TOR pathway, and the Rac-Rho pathway (Reviewed in [35]).

In human cancers, oncogenic altered forms of the proteins N-Ras, H-Ras, and K-Ras aberrantly activate the downstream targets of Ras, leading to uncontrolled proliferation, growth, metabolism, survival, and migration [35]. Mutated Ras proteins are found in 20–30% of human tumors. Significantly, they are often associated with mutations in other genes (such as Myc, tp53, SMAD4), suggesting that mutated Ras alone might not be able to support malignant transformation fully [1,2]. Among these Ras oncogenic versions, the K-Ras protein in which the glycine at codon 12 is mutated into a valine or a serine remains in the GTP-bound constitutive active form. K-Ras is associated with the worst prognosis in many cancer types [4,35,36,37,38]. 

In *Drosophila*, there is only one homolog for N-Ras, H-Ras, and K-Ras, called Ras85D. Similar to its mammalian counterpart, wild type Ras85D is activated by many RTKs and regulates growth [39,40,41,42,43], cell identity [39,41,44,45] and survival [42,46] mainly through the Raf/MAPK pathway [47]. However, Ras85D does not seem to promote proliferation at physiological levels and is likely uncoupled from the PI3K/AKT pathway [40,48]. Similar to K-Ras Codon 12 -mutated proteins, the engineered *Drosophila* *Ras^V12^* allele where valine is substituted with glycine at position 12 produces a constitutively active form of Ras85D [43]. 

*Ras^V12^* MARCM clones in the eye antennal disc grow moderately and overproliferate to form hyperplastic tumors [11] (Figure 1C). In these tumors, *Ras^V12^* increases dMyc levels through Raf/MAPK signaling. Concomitantly and contrary to wild type Ras85D, *Ras^V12^* ectopically activates PI3K/AKT signaling, which might explain why only *Ras^V12^* and not Ras85D expression stimulates proliferation [48]. *Ras^V12^* tumor-bearing larvae can initiate metamorphosis on time. However, most of them die before reaching adulthood, probably because of developmental defects [49]. Interestingly, *Ras^V12^* clones trigger non-autonomous cell death in the surrounding *wild type* (*wt*) cells indicating that there is a sensing mechanism that mediates the detection of transformed *Ras^V12^* cells by *wt* cells [50] (see the section on short-range interaction and cell competition). The few adult escapers display deformed eyes and head structures with necrotic patches [11]. Of note, in the wing disc, *Ras^V12^* cells seem to exhibit some invasive properties as they can migrate through the disc basement membrane. Nevertheless, metalloproteinase production and basement membrane degradation has not yet been demonstrated. *Ras^V12^* transformed cells can also be extruded on the apical side [51]. However, secondary tumor formation has never been observed in this context, indicating that although *Ras^V12^* cells can manage to escape their primary site, they cannot invade other organs and develop into secondary tumors [10,11,52]. Thus, *Ras^V12^* clones in the *Drosophila* imaginal discs form non-invasive benign tumors.

### 2.3. Ras^V12^ Cooperates with the Loss of Cell Polarity to Drive the Formation of Aggressive Malignant Tumors

Malignant tumors are characterized by uncontrolled growth and the ability to invade and destroy other tissues. In the *Drosophila* eye-antennal discs, the concomitant loss of cell polarity (*scrib^−/−^*) and *Ras^V12^* overexpression lead to the formation of aggressive tumors (Figure 1D).

*Ras^V12^; scrib^−/−^* tumor-bearing larvae display a prolonged larval stage. Instead of pupariating and undergoing metamorphosis, they extend their larval life and become giant tumor-bearing larvae. During this time, *Ras^V12^; scrib^−/−^* tumors grow exponentially, invade and completely cover the adjacent larval central nervous system and leg imaginal discs [7,8,10,11,34,52,53,54,55,56]. 

Transformed *Ras^V12^; scrib^−/−^* cells weaken cell–cell adhesion through the downregulation of DE-cadherin [11] and display migratory characteristics such as F-actin enrichment and filopodia formation [7,10,11].

Simultaneously, inside *Ras^V12^; scrib^−/−^* tumors, a subset of cells secrete the metalloproteinase MMP1, which triggers the degradation of the basement membrane [7,8,9,10,11] and allows cells to escape and invade other organs.

In tumor-bearing larvae, *Ras^V12^; scrib^−/−^* cells are found in the ventral nerve cord, 1st and 2nd leg discs, trachea, mouth hook, salivary gland, gut, and fat body. They are highly motile, so that some cells are also able to roam freely in the hemolymph [7,10,11,53].

Strikingly, when given more time to grow thanks to their transplantation into the abdomen of adult flies, *Ras^V12^; scrib^−/−^* tumors overproliferate and form a big mass that kills the host by approximately ten days. This contrasts to *Ras^V12^* or *scrib^−/−^* singly mutated tumors. Transplanted *Ras^V12^; scrib^−/−^* cells retain their migratory characteristic and are found inside the gut wall and the ovarian follicle, a location only reached after several days [11,53]. 

Taken together, these observations demonstrate how the cooperation between *Ras^V12^* overexpression and loss of *scrib* is essential for the development of malignant tumors that proliferate indefinitely and invade other organs (Figure 1). 

The study of the genetic interactions between the two drivers of this model for cooperative oncogenesis has rapidly allowed the identification of key downstream signaling pathways and new concepts, which together explain malignancy.

## 3. Intrinsic Effectors of Malignant Transformation

In this part, we review the intrinsic cues that control *Ras^V12^; scrib^−/−^* cell transformation. We first describe the central yet ambivalent role of JNK signaling in the development of benign vs. malignant tumors. We then highlight the function of the JAK-STAT pathway in this context. Finally, we focus on the role of Salvador-Warts-Hippo signaling.

### 3.1. The JNK Pathway, a Coordinator of Ras^V12^; scrib^−/−^ Malignant Transformation

#### 3.1.1. The Ambivalent Role of JNK Signaling in Benign Tumors vs. Malignant Tumors

High levels of cJun N-terminal Kinase (JNK) signaling have been observed inside *scrib^−/−^* clones [33,57] and *Ras^V12^; scrib^−/−^* clones [7], whereas only basal levels of JNK signaling have been reported in *Ras^V12^* clones [55,58].

Intriguingly, JNK signaling inhibition in *scrib^−/−^* clones leads to the formation of big lethal tumors [10,33,34], whereas in *Ras^V12^; scrib^−/−^* clones, JNK repression gives rise to small non-invasive tumors [9,10].

Thus, JNK signaling has an anti- and a pro-tumorigenic function in *scrib^−/−^* and *Ras^V12^; scrib^−/−^* clones, respectively.

#### 3.1.2. *Drosophila* JNK Signaling Overview

The well-conserved JNK pathway integrates a huge diversity of inputs and promotes various processes such as apoptosis, growth, differentiation inhibition, or migration (reviewed in [59]).

In *Drosophila*, JNK signaling activation results from diverse cues such as oxidative stress, caspases, or binding of the *Drosophila* Tumor Necrosis Factor (TNF) ligand Eiger [33,60,61] to its TNF receptors (TNFR) Wengen (Wgn) [62] or Grindewald (Grnd) [63]. 

In the *Drosophila* eye disc, Eiger overexpression triggers JNK-dependent apoptosis leading to a smaller eye phenotype. This model allowed the characterization of the Eiger-downstream JNK components that are competent in this tissue. Eiger is able to bind to both TNFRs Wgn and Grnd. Whether these receptors possess distinct functions within the pathway is still not clear. However, it has recently been shown that the different binding affinities of Eiger to its receptors govern their internalization trajectory within the cell, which would promote different JNK signaling outcomes [64]. This observation suggests that Wgn and Grnd might have distinct roles in the eye disc. Nevertheless, Egr-induced cell death in the eye disc seems to require both Wgn [62,65] and Grnd [63]. Upon binding to Eiger, the TNFRs activate a kinase cascade including the JNK Kinase Kinase Kinase (JNKKKK) Misshapen (Msn), the JNK Kinase Kinase (JNKKK) Tak1, and the JNK Kinase (JNKK) Hep leading to the final activation of the single JNK, Basket (Bsk). This has long been considered the canonical JNK pathway in the eye disc (Reviewed in [66]). Nonetheless, one might argue that ectopic Eiger expression in the eye disc might lead to aberrant JNK signaling, which does not represent the complete JNK pathway network competent in the eye disc. Notably, more studies in other tissues and contexts have further expanded the list of JNK signaling upstream components in *Drosophila* at every level of the pathway [63,67]). 

Once activated, Bsk phosphorylates several transcription factors (TFs). The most famous ones are the TFs Kayak (Kay aka Fos) and Jun-related antigen (Jra), which heterodimerize to form the so-called AP-1 complex. Transcriptional targets include the proapoptotic genes *reaper* and *hid*, the negative JNK regulator *puckered* (*puc*), the JNKKKK *msn*, the matrix metalloproteinase *MMP1*, the integrin-associated gene *paxillin*, the JAK-STAT ligands *upd1*, *upd2* and *upd3* and the Wnt signaling ligand *wingless* (*wg*) (reviewed in [59].

#### 3.1.3. JNK Signaling Functions in *scrib^−/−^* Versus *Ras^V12^; scrib^−/−^* Tumors

Given the antagonist observations upon JNK signaling inhibition in *scrib^−/−^* versus *Ras^V12^; scrib^−/−^* clones, many studies have contributed to deciphering the mechanisms that control JNK pro- and anti-tumorigenic functions.

**Figure 2:**
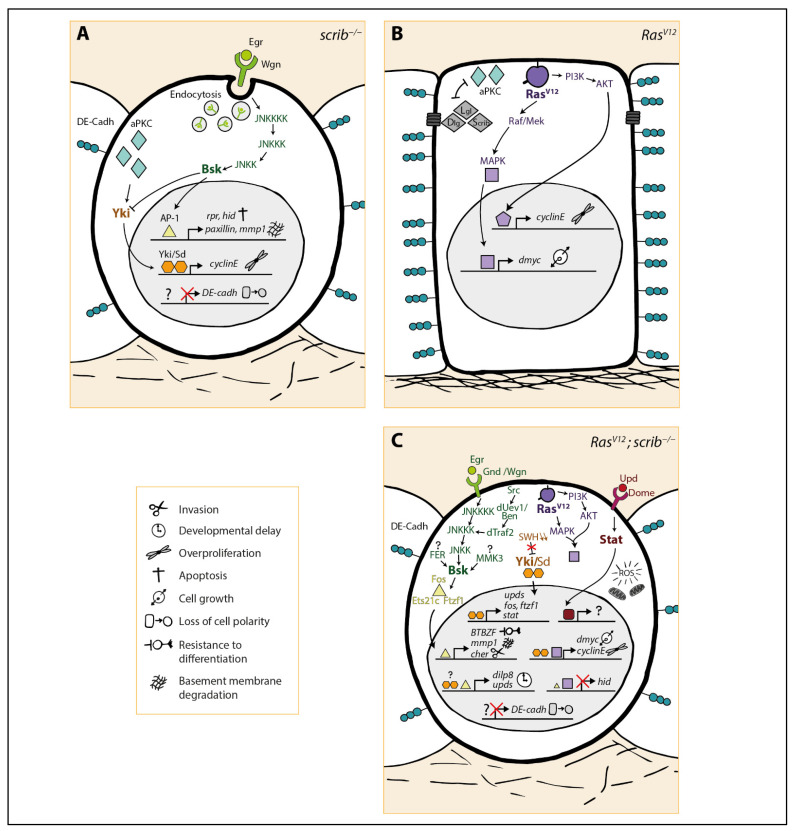
Cooperative oncogenesis results from the atypical interplay of various signaling pathways that display several tumor cancer hallmarks. (**A**) In *scrib^−/−^* benign tumors, the JNK pathway (green) has an antitumorigenic function. It is activated by the TNF Eiger, which binds to the TNFR Wengen (Wgn). Elevated endocytosis levels contribute to activating the Eiger/Wgn complex that activates a phosphorylation event cascade involving successively the JNKKKK, JNKKK, and JNKK. Ultimately, the JNK Basket is phosphorylated and activates its downstream AP-1 TFs effectors. AP-1 TFs stimulates the expression of the proapoptotic factors rpr and hid, which execute apoptosis a well as paxillin and mmp1, which leads to basement membrane degradation. Simultaneously, the loss of cell polarity initiated by the loss of function of *scribble* provokes aPKC (blue) signaling impairment, which results in the activation of the SWH target Yki (orange). Yki together with Sd activates the expression of cyclinE and causes overproliferation. Finally, DE-cadherin expression decreases, contributing further to the loss of cell polarity phenotype. (**B**) In *Ras^V12^* hyperplastic tumors, the Scrib (grey) and aPKC (blue) apical complexes usually are localized close to the adherens junction and in the apical part, respectively. The aberrantly active *Ras^V12^* stimulates the downstream Raf/Mek/MAPK and PI3K/AKT pathways (purple) responsible for cyclinE (overproliferation) and dmyc (cell growth) overexpression, respectively. (**C**) In the *Ras^V12^; scrib^−/−^* neoplastic tumors, JNK is protumorigenic. The upstream JNK signaling is not fully explored in the eye-antennal disc tumors yet. It seems to involve Eiger binding to Grnd mainly (and Wgn), which further activates the JNKKKK Msn, the JNKKK Tak1, the JNKK Hep, and Bsk. A parallel Src-dUev1/Ben/dTraf2 axis elevates the activity of Hep upstream of Bsk. The novel JNKK MMK3 and the nonreceptor tyrosine kinase FER might also contribute to Bsk activity. Bsk triggers the activation of the TFs Fos, Ets21c, and Ftfz1, which upregulate the expression of the BTBZF chinmo and abrupt (resistance to neuron differentiation), mmp1 and cher (basement membrane degradation and invasion), as well as dilp8 and upd (developmental delay). Whether they also elevate hid transcription is unknown. In parallel, lowering of SWH signaling leads to the activation of Yki (orange). Together with Sd, Yki activates the expression of dmyc (cell growth), cyclinE (proliferation), dilp8 and upd (developmental delay, not demonstrated) and of some other signaling components such as upd and stat (JAK-STAT pathway) as well as fos and ftzf1 (JNK pathway). *Ras^V12^* activates the Raf/Mek/MAPK and PI3K/AKT pathways, contributing to the expression of dmyc (cell growth) and cyclinE (proliferation). It also inhibits hid expression and Hid activity, protecting *Ras^V12^; scrib^−/−^* cancer cells from apoptosis. Finally, tumor-secreted Upds activate the JAK-STAT pathway in an autocrine manner and contribute to ROS production and tumor growth through an unknown mechanism.

In *scrib^−/−^* clones, JNK is active in most cells and induces apoptosis [10,33,34] most probably through the upregulation of the apoptosis-triggering genes *reaper* and *hid* [60,68]. Interestingly, in these dying tumor cells, high levels of endocytosis are observed, which cause the relocalization of Eiger/Wengen complexes from the plasma membrane to endosomal vesicles. This step is required for JNK activation and induction of apoptosis [33]. Simultaneously, JNK promotes the expression of the integrin-associated protein Paxillin and the expression of MMP1, leading to basement membrane degradation [7,33,34,69]. However, *scrib^−/−^* cells most likely die before acquiring invasive properties, as no migrative behavior has been observed in these cells. JNK activation inside *scrib^−/−^* cells also controls the inhibition of photoneuron differentiation [33,34]. In summary, in *scrib^−/−^* benign tumors, the JNK anti-tumorigenic effect consists mainly in the induction of apoptosis while JNK also contributes to differentiation inhibition and degradation of the basement membrane (Figure 2A). It is worth mentioning that JNK signaling does not promote the CyclinE-mediated overproliferation phenotype or cell morphology alterations [69]. 

In *Ras^V12^; scrib^−/−^* tumors, JNK signaling is activated in patches of cells within the tumor and migrating cells that left the primary site [7,9,69]. In these cells, JNK promotes the upregulation of MMP1 and the actin cross-linking protein Cheerio necessary for basement membrane degradation and cytoskeleton remodeling, respectively, together leading to tumor cell migration [7,9,10,70]. It also promotes Paxillin expression, whose function has not been further investigated [69]. Surprisingly, it has been reported that JNK activation in this context is dependent on Kay, but not on its conventional partner Jra [10]. Kay then triggers the expression of the TFs Ftz-f1 and Ets21c (which it associates with), which are essential mediators of the JNK-driven malignancy [54,71,72]. JNK signaling also represses photoneuron specification through the BTB Zinc finger proteins (BTBZF) Chinmo and Abrupt [73]. The JNK target Cheerio also contributes to differentiation inhibition while enhancing proliferation [70]. The switch from anti- to protumorigenic function of JNK lies in the inability of *Ras^V12^; scrib^−/−^* cells to undergo apoptosis although caspases are activated [74]. This is due to the fact that *Ras^V12^* both downregulates hid [75] and represses Hid activity [42]. At the same time, *Ras^V12^* boosts proliferation through the PI3K pathway and Myc [76]. To conclude, in *Ras^V12^; scrib^−/−^* cells, JNK promotes invasion (basement membrane and cell motility), differentiation inhibition, and most likely proliferation, but it cannot trigger apoptosis (Figure 2C).

Because JNK signaling is such a strong driver of *Ras^V12^; scrib^−/−^* neoplasia, it is crucial to understand how the pathway is activated.

#### 3.1.4. JNK Signaling Upstream Mechanisms in *scrib^−/−^* Versus *Ras^V12^; scrib^−/−^* Tumors

Whereas Eiger has been reported to be the primary activation signal upstream of JNK in *scrib^−/−^* cells [33], several studies report a more complex and diversified upstream network for JNK signaling *Ras^V12^; scrib^−/−^* cells.

*Ras^V12^; scrib^−/−^* cells have long been thought to activate JNK signaling only through Eiger as *eiger* loss of function impairs tumor growth. Both Grnd and Wgn seem to be involved [7,55,63,77], although Wgn appears to have a minor contribution to JNK signaling activation compared with Grnd [55,77]. Most probably because of technical issues, the other components leading to JNK activation have only been validated in *Ras^V12^; lgl^−/−^* eye disc clones (who display a similar neoplastic phenotype as *Ras^V12^; scrib^−/−^* tumors [11]). It consists of Hep, Tak1, and dTraf2 (an adapter protein that activates Tak1) [55]. Several additional players have recently been identified. For instance, the novel JNKK MKK3 (Map Kinase Kinase 3 aka Licorne) positively modulates JNK activation in parallel of Hep and promotes invasion of tumor cells [78]. Additionally, the nonreceptor tyrosine kinase Scr42a activates the dUev1/ben ubiquitin-conjugating enzymes complex upstream of Traf2 and further contributes to JNK signaling [79,80]. Interestingly, overexpression of Src42a in *Ras^V12^* tumors promotes neoplastic growth through JNK signaling and PI3K signaling, confirming the link between Src42a and JNK signaling in tumorigenesis [81]. Of note, another non-receptor protein tyrosine kinase called FER has also been identified in the wing disc, where it directly phosphorylates Bsk and promotes migration of *scrib knock-down* (*scrib^KD^*) cells [82]. Similarly, the JNKKK Wallenda (Wnd) binds to Rho1 in order to modulate migration in the same system. [63,67] (Figure 2C). Whether FER or the Wnd/Rho1 complex also contribute to neoplasia in the *Ras^V12^; scrib^−/−^* eye disc tumors still need to be elucidated. Notably, the nature of the activating signal upstream of MKK3, Src42a, FER, or Wnd has not yet been addressed. Because in other systems, JNK signaling can integrate cues from various processes such as oxidative stress, novel JNK modulators within *Ras^V12^; scrib^−/−^* tumor cells might be uncovered in the future [77,83].

Finally, in *Ras^V12^* clones, the cells of the edge display basal levels of JNK signaling. Thus, although competent for JNK activation, *Ras^V12^* cells seem to refrain from activating JNK in all cells. Autophagy and ROS inhibition are thought to lower JNK signaling in this context [84]. Notably, JNK ectopic activation turns *Ras^V12^* benign tumors into invasive tumors [10]. Similarly, in human, JNK, and H-RasV12 also cooperate to promote the invasiveness of human mammary cells. This is of great relevance as Her2+ breast cancer is characterized by both Ras/Erk signaling activation and JNK signaling signature [58]. 

Taken together, these studies reveal that JNK signaling lies at the heart of the cooperation between *Ras^V12^* and the polarity deficiency to drive malignancy in *Drosophila* and that it seems conserved in human cancer.

### 3.2. JAK-STAT: A Cytokine-Triggered Signaling Pathway That Amplifies Tumor Growth

The evolutionary conserved JAK-STAT (Janus Kinase and Signal Transducer and Activator of Transcription) has been implicated in processes as diverse as tissue homeostasis control, sex determination, or immune response. In *Drosophila*, the JAK-STAT pathway is canonically activated by the binding of the cytokines Unpaired (Upd) 1, Upd2, or Upd3 to the dimerized Dome receptor. Upon binding, Dome triggers the activation of the JAK protein Hopscotch (Hop) anchored to its cytoplasmic domain. Hop autophosphorylates itself and phosphorylates Dome to provide free access for the pathway effector STAT92E (Signal Transducer and Activator of Transcription at 92E) to the docking sites on Dome. In this way, inactive STAT92E is recruited and phosphorylated. Subsequently, active STAT92E forms homodimers which translocate to the nucleus. There, STAT92E binds to DNA palindromic sequence. It recruits transcriptional activators and promotes the transcription of many targets, including cyclinD, antimicrobial peptides, or proto-oncogenes such as draf (Reviewed in [85]). 

In *Ras^V12^; scrib^−/−^* tumors, JAK-STAT signaling is widely activated, and the three Upd ligands are overexpressed (Figure 2C). Inhibition of the JAK-STAT pathway by the overexpression of a dominant negative form of Dome (DomeDN) drastically reduces tumor growth and abrogates invasion. Interestingly, loss of function of *stat92e* only mildly impairs tumor growth and invasion, suggesting that other uncharacterized JAK downstream effectors might contribute to malignant transformation [86]. Moreover, overexpression of Upd in *Ras^V12^* clones is sufficient to induce massive tumor growth and invasion [86], demonstrating that *Ras^V12^* can also cooperate with Dome-JAK-STAT to drive malignancy. This observation prompted a few labs to investigate whether the JAK-STAT pathway is linked to JNK signaling in *Ras^V12^; scrib^−/−^* tumors.

Indeed, in wing discs homozygous for *scrib^−/−^* or bearing *dlg^KD^* clones, JNK signaling promotes JAK-STAT activation [86,87]. Moreover, in *Ras^V12^, dlg^KD^* eye disc tumors (who display a similar neoplastic phenotype as *Ras^V12^; scrib^−/−^* tumors [11]), the JNK downstream effector Fos associates with the TF Ets21c to upregulate upd1 [71]. Thus, although not fully demonstrated, the common view is that JNK signaling activates the JAK-STAT pathway in *Ras^V12^; scrib^−/−^* eye disc tumors through autocrine Upd production. 

It has also been demonstrated that JAK-STAT activation in the *Ras^V12^; scrib^−/−^* tumors increases ROS levels necessary for tumor growth [54,74]. Nevertheless, the identity of the STAT92E targets in this tumor model remains largely unexplored.

### 3.3. The Salvador–Warts–Hippo Pathway: When the Brake Breaks

The Salvador–Warts–Hippo (SWH) pathway coordinates mechanical inputs with organ homeostasis. It integrates various parameters such as cell polarity, cell contact, and G-protein coupled receptor signaling to fine-tune cell proliferation and survival. Defects of the SWH pathway are associated with poor prognosis in many cancers and are thought to help cancer cells escape cell ‘contact inhibition’ [88,89].

Although the main target of the SWH pathway, YAP (Yes-Associated Protein), was identified first in yeast and mammals, its functions and regulation remained unclear until the discovery of its *Drosophila* ortholog Yorkie (Yki). Indeed, the upstream regulators of Yki were identified first in *Drosophila*, contributing greatly to the understanding of the conserved regulation and functions of YAP in mammals both during development and tumorigenesis (reviewed in [89]).

The core of the SWH pathway consists of three interacting proteins: Hippo, Salvador, and Warts. The Hippo (Hpo) Serine Threonine kinase phosphorylates its adaptor protein Salvador (Sav) to form an activated Hpo-Sav complex. This complex phosphorylates and activates the Serine Threonine kinase Warts (Wts) and its adaptor protein Mob As Tumor Suppressor (Mats). Together, they form the activated Wts–Mats complex, which inactivates through phosphorylation the Yes-associated protein ortholog Yki. Yki is a transcriptional co-activator that, when phosphorylated, is kept in the cytoplasm and sent for degradation through its association with 14-3-3 proteins. When its phosphorylation state is low, Yki enters the nucleus. There, it associates with the transcription factor Scalloped (although other Yki partner TFs exist) and activates the transcription of many target genes involved in cell proliferation, like *cyclinE*, and in cell survival, like *diap1* (Death-associated Inhibitor of Apoptosis 1).

Thus, by repressing Yki activity, the SWH pathway is a central negative regulator of proliferation and survival.

Upstream activators of the SWH pathway are key players of cell junction and adhesion. For instance, a subapical complex composed of the FERM domain proteins Merlin and Expanded and the adaptor protein Kibra integrates cell contact and polarity signals. Consequently, this complex binds to the core components of the SWH and promotes sequestration of Yki in the cytoplasm. Similarly, the proto-cadherin Fat modulates the pathway positively.

*Scrib^−/−^* clones in the eye disc display an overproliferation phenotype that is not controlled by JNK signaling. Instead, overproliferation results from the inhibition of the SWH pathway by aberrant aPKC activity due to *scrib* loss of function [26,90] (Figure 2A). Similarly, SWH downregulation is also observed in *lgl^−/−^* clones in the eye disc and the wing disc and provokes CyclinE and Diap1 upregulation [26,91]. Unexpectedly, suppression of JNK signaling within *scrib^−/−^* eye disc clones enhances Yki elevation, suggesting that JNK restricts tumor growth through SWH signaling [90,92]. Thus, in *scrib^−/−^* eye disc clones, Yki activity, promoted by aPKC and repressed by JNK, stimulates tumor cell proliferation. Contrary to the eye disc tumors, in *lgl^KD^* and *dlg^KD^* wing disc tumors, Yki-induced proliferation is promoted by JNK signaling [93]. This significant difference underlines the importance of being cautious while comparing the loss of cell polarity-induced tumors that develop in different tissues.

In *Ras^V12^; scrib^−/−^* tumors, high Yki/Sd activity levels promote tumor proliferation and invasion [70,90,94,95] (Figure 2C). Contrary to *scrib^−/−^* clones, JNK inhibition, does not affect the expression of Yki targets in *Ras^V12^; scrib^−/−^* tumors [73,92], although the JNK target Cher is necessary for the expression of the Yki target expanded [70]. Unexpectedly, targets of Sd within *Ras^V12^; scrib^−/−^* tumors are predicted to include the main effectors of the JAK-STAT pathway (*stat92E*) and JNK pathway (*fos*, the Jra partner *atf3* and *ftz-f1*) [95] suggesting that Yki might instead act upstream of both JNK and JAK-STAT signaling. Indeed, Yki inhibition in *Ras^V12^; scrib^KD^* wing disc tumors suppressed JNK signaling and tumor growth [94]. This is puzzling because JNK signaling is upstream of the SWH pathway in *scrib^−/−^* eye disc clones. Moreover, several examples of SWH inhibition by the JNK pathway have been reported in both wild type and tumor-bearing wing discs [93,96] and eye discs [97]. Finally, JNK signaling activation in *Ras^V12^* eye disc clones increases Yki activity [97,98], reinforcing the idea that JNK signaling is upstream of Yki. Further work would be required to address the exact relationship between the JNK pathway and the SWH pathway. aPKC might be another potential inducer of Yki activity in *Ras^V12^; scrib^−/−^* tumors, given its tumor-promoting function in *scrib^−/−^* clones. However, aPKC inhibition does not impact *Ras^V12^; scrib^−/−^* tumor growth and invasion [69]. Recently, it has been demonstrated that in presence of *Ras^V12^*, JNK inhibits Wts activity and promotes Yki activity in the eye disc. However, this mechanism has not yet been demonstrated in *Ras^V12^; scrib^−/−^* tumors [99]. 

To conclude, the proliferation, differentiation inhibition, survival, and invasion of *Ras^V12^; scrib^−/−^* cells are governed by a cocktail of three main signaling pathways: the JNK, the JAK-STAT, and the SWH pathway (Figure 2). Chromatin profiling and transcriptomic analysis of *Ras^V12^; scrib^−/−^* eye disc tumors have validated the importance of these three pathways for reshaping the malignant cells genomic and transcriptomic landscapes. Remarkably, they conclude that less than ten transcription factors account for the neoplastic transformation of *Ras^V12^; scrib^−/−^* [95,100]. The JNK transcription factor Fos and the JAK-STAT transcription factor Stat92E are predicted to target 70% and 10% of the tumor-induced regulatory regions, respectively [100]. Nevertheless, the hierarchy and interdependency between them remain unresolved.

Because *Ras^V12^; scrib^−/−^* tumor cells express the secreted cytokines Upd1, Upd2, and Upd3, as well as the TNF homolog, Eiger, several studies have focused on how the tumor cells affect their neighboring wild type cells.

## 4. Microenvironmental Cells Control Tumor Growth in Various Ways

The *Drosophila* single insult *scrib^−/−^* and the *Ras^V12^; scrib^−/−^* cooperative oncogenesis models have both been instrumental in understanding how the microenvironment affects cancer progression. Indeed, both anti- and pro-tumorigenic local interactions have been described. Here we will review the basic principles and examples of how the microenvironment can assume both these roles.

### 4.1. Scrib^−/−^ Model—A Tumor Suppressive Role of the Microenvironment through Cell Competition

Landmark studies in *Drosophila* have identified a critical “surveillance mechanism” termed cell competition (reviewed in the same Issue [101]). Patches of cells with detrimental mutations, called losers, are actively eliminated by wild type neighbors at the clonal border, termed winner cells [60,102]. This phenomenon ensures that abnormal cell populations do not colonize and dominate the tissue and were later shown to be crucial for suppressing polarity-deficient tumors [34]. For instance, *scrib^−/−^* clones are efficiently removed from the epithelium during development, so that very few cells survive to adulthood. Strikingly, when the whole tissue is mutant for *scrib* or when surrounding cells are genetically ablated, apoptosis in *scrib^−/−^* cells is suppressed, and they develop into malignant tumors [34,103]. This observation demonstrates the key tumor-suppressive role of *wt* neighboring cells.

How do *wt* cells eliminate their oncogenic insulted neighbors? Indeed, the elimination of these tumors is genetically encoded by a set of molecular programs, which results in their commitment to the loser status, impaired proliferation, induced entosis, and ultimately death.

**Figure 3:**
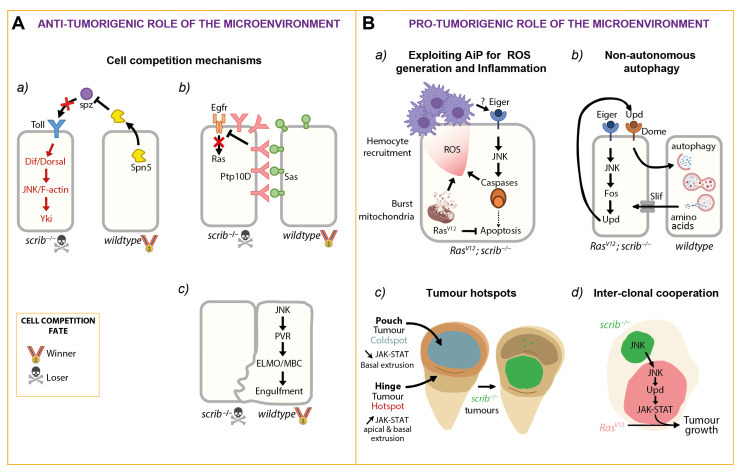
Local microenvironment interactions can act as suppressors or be exploited for tumor growth. (**A**) Anti-tumorigenic role of the microenvironment. *scrib^−/−^* tumors are actively eliminated by the neighboring cells through cell competition. Several mechanisms are put in place by the winner cells, the microenvironment, to restrict the growth and remove the loser cells, the *scrib^−/−^* tumors. (**a**) The expression of an inhibitor of the toll ligand Spz, Spn5, is critical in the microenvironment to maintain the loser status of *scrib^−/−^* tumors. Upon non-autonomous loss of spn5, Toll signaling, together with JNK and high F-actin levels, can elevate yki activity, an inducer of the winner status (shown in red). (**b**) Upon tumorigenesis, *wt* cells communicate with the tumor cells through the Sas-Ptp10D signaling axis to inhibit Egfr-Ras signaling, a pathway that can potently cooperate with polarity deficiency for malignancy. (**c**) Activation of JNK in the *wt* cells activates an engulfment program through PVR-ELMO/MBC which leads to the entosis of the *scrib^−/−^* tumors and their death. (**B**) Pro-tumorigenic role of the microenvironment. In a cooperative *Ras^V12^; scrib^−/−^* scenario, the microenvironment is exploited to fuel tumor growth. (**a**) Caspase apoptotic induction is inhibited by *Ras^V12^*; however, apoptotic-independent functions still remain active. *Ras^V12^; scrib^−/−^* tumors exploit the mechanism behind apoptosis induced proliferation. Caspases, and potentially impaired mitochondria, can induce high levels of ROS, which triggers hemocyte recruitment. This mounts an inflammatory response that leads to higher JNK levels. (**b**) NAA is induced in the *wt* cells surrounding *Ras^V12^; scrib^−/−^* tumors, through tumor autonomous JNK and the autocrine activation of JAK-STAT through Upd and Dome. Downregulation of the transporter slif in *Ras^V12^; dlg^−/−^* showed that these tumors are dependent on exogenous nutrient sources, potentially released from the neighboring *wt* cells through NAA. (**c**) Areas that are nurturing or hindering for tumor growth, termed tumor “hotspots” and “coldspots”, respectively, have been identified in the wing disc. The tumor hotspot, which is in the wing disc hinge, was found to have high levels of JAK-STAT, a pathway critical for the *Ras^V12^; scrib^−/−^* tumor growth, and apical extrusion which promotes tumor cell survival. (**d**) The *Ras^V12^; scrib^−/−^* cooperative scenario is not restricted to an intraclonal setting. Neighboring *scrib^−/−^* clones to *Ras^V12^* tumors has been found to be sufficient to elevate JNK and JAK-STAT in *Ras^V12^* tumors, and to trigger their large overgrowth and invasion.

As described above, the stress pathway JNK is activated in *scrib^−/−^* clones, where it triggers apoptosis [33,34]. JNK additionally downregulates Yki activity preventing overproliferation [92]. The dampening of Yki activity is also achieved through the serine protease inhibitor Serpin 5 (Spn5) [104] (Figure 3A(a)). Spn5 inhibits Toll signaling by preventing the activation of the Toll ligand Spaetzle [104]. spn5 knock-down in the microenvironment provokes high levels of toll signaling in *scrib^−/−^* clones (Katsukawa et al., 2018). Ectopic activation of Toll signaling turns losers into winners and induces the death of *wt* microenvironmental cells [104], a phenomenon called supercompetition [104]. These *scrib^−/−^*, Toll-high converted winners also have upregulated Yki activity [104], a known trigger of supercompetitor status [105]. Thus, the lowering of Yki activity causes the elimination of polarity deficient clones both by toning down the tumor cell proliferation and preserving the tumor suppressive microenvironment.

An exciting question emerging from these studies is: how do healthy epithelial neighbors sense the presence of transformed cells and execute their elimination? 

To restrict *scrib^−/−^* tumor growth, the binding of the Receptor tyrosine phosphatase Ptp10D in tumor cells to its surface ligand-protein Sas in *wt* neighboring cells is critical (Figure 3A(b)). Interestingly, when in contact, the *scrib^−/−^* cells and their healthy neighbors perform an apical-lateral translocation of the receptor Ptp10D and the ligand Sas, respectively. Failure in establishing this Ptp10D-Sas crosstalk results in elevated Epithelial Growth Factor Receptor (Egfr) signaling activity, which then cooperates with JNK to drive overgrowth of *scrib^−/−^* tumors [106], reminiscent of the *Ras^V12^; scrib^−/−^* oncogenesis. In line with this finding, in mammals, the EGFR is negatively modulated through its dephosphorylation by the orthologs of Ptp10D [107,108]. 

JNK signaling in the microenvironment is also part of the crosstalk between the tumor cells and their neighbors. Indeed, JNK activity is not only upregulated in the *scrib^−/−^* tumor but also in the surrounding *wt* cells [57]. Surprisingly, this JNK activation in the microenvironmental cells is required for tumor suppression by entosis (defined as the engulfment of tumor cells by *wt* cells) [109]. In these *wt* neighbors, JNK upregulates the receptor protein-tyrosine kinase Pvr (PDGF- and VEGF-receptor related) and its downstream effectors, the GTPase regulators ELMO (engulfment and cell motility) and Mbc (Myoblast city) [57] (Figure 3A(c)). ELMO and Mbc are thought to form a guanine-nucleotide exchange factor complex which positively regulates Rac1 [110], which is believed to stimulate cytoskeletal dynamics. This should then orchestrate loser cell phagocytosis and ultimately engulfment, and interestingly has been shown to be involved in another cell competition context [111]. Of note, JNK activation in *wt* border cells does not induce apoptosis [57], contrary to the tumor compartment. A possible explanation for this observation is that Yki activity also seems upregulated in the surrounding neighbors [92]. Then, one of the main targets of Yki, *diap1*, might promote survival of the *wt* cells at the border of the tumor despite high JNK activity. Another lead for explaining the survival of border untransformed cells could be the generation of ERK waves. Indeed, it has been shown recently that ectopic induction of apoptosis in epithelia composed of *wt* cells triggers ERK waves in the surrounding non-dying cells, which supports survival through inhibition of caspases [112]. Therefore, it is plausible that a similar mechanism takes place in the surrounding *wt* cells of a *scrib^−/−^* dying tumor. Future experiments will test whether these phenomena could trigger the conditional survival of the neighboring cells to the JNK stress pathway. To conclude, microenvironmental cells restrict *scrib^−/−^* tumor growth by lowering their proliferation through the Ptp10D-Sas axis, eliminating tumor cells by entosis through Pvr signaling, and actively surviving to aberrant JNK signaling through Yki elevation. 

Overall, these studies showcase the critical importance of the local host cells, where several genetic programs are put in place to halt tumor progression.

### 4.2. Ras^V12^; scrib^−/−^ Model—A Microenvironmental Pro-Tumorigenic Role Switch in a Cooperative Scenario

#### 4.2.1. Undead *Ras^V12^; scrib^−/−^* Tumor Cells Exploit Apoptosis-Induced Proliferation, ROS, and Inflammation

Upon the elimination of unfit cells from the tissue, surrounding surviving cells overproliferate to preserve tissue homeostasis. This safeguarding mechanism is known as apoptosis-induced proliferation (AiP). The apoptosis of a cell within a *Drosophila* imaginal epithelium leads to the generation of reactive oxygen species (ROS) and mitogens, which stimulate the proliferation of the neighboring cells [113]. Indeed, AiP is thought to be responsible for the normal appearance of the adult eyes in which *scrib^−/−^* tumors formed and were progressively eliminated (Wu et al., 2010b). In detail, the downregulation of Upd and Stat92E in the *scrib^−/−^* tumor and its microenvironment, respectively, account for a visible reduction of the adult eye. This observation shows that a JAK-STAT communication axis between both compartments is essential to execute AiP and replenish tissue cell loss [86].

Nevertheless, the large majority of our knowledge underlying the molecular mechanisms behind AiP comes from the study of “undead” cells. In this model, cells are fated genetically for cell death through the upregulation of the pro-apoptotic factors Reaper and Hid. Typically, they trigger the activation of the initiator caspase Dronc through Diap1 inhibition. Then, Dronc activates the effector Caspases Dcp-1 (Decapping protein 1) and DriCE (Death-related ICE-like caspase), leading to apoptosis. Upon coexpression of the effector caspase inhibitor, p35, apoptosis cannot proceed, leaving the cell in an undead state [113].

In “undead” cells, Dronc can activate the production of extracellular ROS through Duox, which alarms hemocytes to mount an inflammatory response through Eiger. This is suggested to promote JNK activation in epithelial cells, mitogen expression, and AiP [114]. 

Interestingly, *Ras^V12^; scrib^−/−^* tumors behave much like the classical “undead” cells and exploit a similar mechanism [74]. Strikingly, *Ras^V12^; scrib^−/−^* tumors strongly accumulate ROS, most probably because of impaired mitochondrial function [54] and Caspase activity [74] as well as they attract hemocytes (Figure 3B(a)). Upon the downregulation of both extra and intracellular ROS production, JNK signaling is inhibited, tumor growth is largely impaired, and adult survival is restored. These findings demonstrate how critical ROS are for malignant transformation [74]. Overall, this study suggests tumors hijack the AiP mechanisms for ROS generation through Caspases to elevate JNK levels [74], which is essential to drive malignancy in this tumor model. 

#### 4.2.2. Non-Autonomous Autophagy Fuels Tumor Growth

Due to their aggressive proliferative growth and migratory behavior, neoplastic tumors have a high anabolic demand. Neighboring healthy cells could constitute a valuable source of nutrients. *Ras^V12^; scrib^−/−^* tumors induce autophagy in the microenvironment [54] (Figure 3B(b)). Autophagy is a process by which cells can degrade macromolecular components in the lysosome to recycle building blocks in nucleotides, sugars, amino acids, and fatty acids for anabolic or energetic demands. When autophagy is inhibited in the microenvironment, it strongly reduces tumor growth and malignancy *in vivo* [54,115]. Downregulation of an amino acid transporter Slimfast (Slif) limits the growth of a related cooperative model, *Ras^V12^; dlg^−/−^* [54], suggesting that these tumors are highly dependent on nutrients from their environment. In line with this finding, pancreatic tumors are provided amino acids via autophagy from their neighboring stellate cells [116]. In *Ras^V12^; scrib^−/−^* eye disc tumors, non-autonomous autophagy (NAA) was shown to depend on the JNK signaling in the tumor and the autocrine activation of JAK-STAT through the Upd cytokines and Dome receptor [54]. However, the exact trigger and sensor for NAA in the microenvironment and whether nutrient mobilization is the sole role of autophagy in this model remains to be discovered. 

Overall, although autophagy has been classically regarded as a tumor-suppressive mechanism, this study reinforced the idea that it can play an ambivalent role in cancer [117]. The capacity to be pro-tumorigenic was dependent on an oncogenic cooperation network, highlighting the importance of the tumor mutational context when designing therapeutic approaches to target this pathway. 

#### 4.2.3. Tumor Hotspots

A central hypothesis in the field of cancer research is the existence of pro-tumorigenic niches: specific areas in tissues or organs that are more favorable for initial tumor growth or metastasis. Understanding the exact molecular nature of why some zones are more nurturing is of high interest since this could allow the prediction of oncogenic target sites and early cancer detection. 

Interestingly, a study in the wing imaginal disc identified a tissue region where large dysplastic *lgl^KD^* and *scrib^KD^* tumors (defined as abnormally growing lesions) were consistently generated, as opposed to another area where these overgrowths were not observed. These zones where thus designated tumor “hotspots” and “coldspots”, and they were found in the dorsal hinge and the disc pouch, respectively [118] (Figure 3B(c)). 

These areas have two critical differences. Inflammatory pro-proliferative JAK-STAT signaling is active during development in the dorsal hinge. Furthermore, upon the generation of *lgl^KD^* and *scrib^KD^* clones, in both regions, extrusion of apoptotic cells to the basal side of the disc was observed. However, only in the hotspot zone, polarity deficient clones extruded apically and generated dysplastic tumors [118]. 

Indeed, Stat92E was found to be required in the apically extruded clones in the hotspot zone to generate dysplastic large clones. This exciting result shows that endogenous, “pre-patterned” tissue signaling programs can cooperate with tumor cells and drive their growth. Furthermore, through the knock-down of the cytoskeletal regulator rho guanine nucleotide exchange factor 2 (rho-gef2) it is possible to induce both basal and apical extrusion of *lgl^KD^* and *scrib^KD^* clones in the pouch region. However, this is not sufficient to induce large dysplastic tumors in the pouch. This is only possible upon ectopic induction of JAK-STAT signaling combined with induced apical extrusion of clones in the coldspot zone [118].

To conclude, the endogenous tissue signaling and the polarity of cell extrusion can vary spatially, providing certain areas with increased risk for tumorigenesis. This study demonstrated an important principle, where cooperative scenarios promoting tumor growth can be generated through the interaction of gene mutations and additionally endogenous wildtype tissue programs installed during development and potentially homeostasis.

#### 4.2.4. Interclonal Cooperation

Classically, the microenvironment is classified as all non-transformed cells surrounding the tumor and the extracellular matrix. Additionally, in the vicinity of a tumor cell, other cancer cells with similar or different mutant backgrounds can exist and potentially establish a communication axis to influence each other’s fate. *Drosophila* offers a platform for the dissection of the molecular underpinnings behind such clonal interactions and the opportunity to identify new basic molecular principles.

A previous study examined the behavior of separate *Ras^V12^* and *scrib^−/−^* clones generated individually in the same eye antennal disc (*Ras^V12^//scrib^−/−^*) [86]. Strikingly, these clones cooperate, resulting in strong overgrowth and invasion into the ventral nerve cord (Figure 3B(d)). These tumors are composed mainly of *Ras^V12^* cells. Like in double mutant *Ras^V12^; scrib^−/−^* tumors, a reporter for JAK-STAT showed high activity in tumors, and the upd ligands were highly expressed. Inhibiting JAK-STAT signaling through Dome^DN^ expression in *Ras^V12^* cells facing *scrib^−/−^* cells completely inhibited tumor growth and invasion, demonstrating the critical role of inflammatory pro-proliferative signals in this interclonal cooperation scenario. Furthermore, inhibiting JNK in *Ras^V12^* cells, but not in neighboring *scrib^−/−^* clones results in a substantial reduction in growth and inhibition of malignancy. Moreover, concomitant expression of Upd and Bsk^DN^ (a dominant negative form of Bsk) in *Ras^V12^* clones recapitulated the phenotype observed in *Ras^V12^//scrib^−/−^*. *Ras^V12^* clones have low JNK levels, and thus these results suggest that JNK signaling can propagate from *scrib^−/−^* to *Ras^V12^* tumors within the same disc. This then drives the upregulation of JAK-STAT and sequentially the overgrowth and malignancy of *Ras^V12^* clones [86].

To prove that JNK signaling can propagate, the authors carried out an elegant experiment in the wing imaginal disc. Here, the inhibitor of JNK, Puckered, was overexpressed medially in the patched domain. Wounding, a known trigger of JNK, was then performed in only one side of the disc. Interestingly, as compared to control, JNK activity did not propagate beyond the patched stripe overexpressing Puckered [86]. This demonstrated that JNK indeed propagates and that this propagation requires JNK activity. However, others have shown that JNK propagation to the host cells from *dlg^−/−^* tumors are independent of JNK [57]. Possibly, in a tumorigenic vs. wounding scenario, additional mechanisms are put in place to trigger a host response, but this signal still remains undiscovered.

Thus, non-autonomous tumor interactions are not restricted to the *wt* neighbors but can additionally be established with other tumors in their vicinity. Furthermore, Wu et al., underlined that oncogenic cooperation does not necessarily need to be generated within the same cell population but can be established from individually adjacent compartments to achieve a malignant outcome. 

## 5. Long-Range Interactions between the Tumor and Its Host Are Critical for Tumor Growth and Invasion

Tumor cells not only communicate with their direct neighbors but also initiate cross-talk with distant organs. This section reviews how the *Ras^V12^; scrib^−/−^* tumors lessen ecdysone production in the prothoracic gland to halt larval to pupal transition-pupation. Then we focus on the enigmatic contribution of the inflammation response mediated by tumor-associated immune cells and NFκB signaling in the tumor. Finally, we discuss the contribution of tumor-induced organ wasting.

### 5.1. The Tumor Halts the Larval Development and Gains More Time to Grow

*Ras^V12^; scrib^−/−^* tumor-bearing larvae linger, meaning that instead of pupariating at day 6 and undergoing metamorphosis into an adult at day 10, they extend their larval life up to 15 days. During this aberrant larval period, tumor-bearing larvae keep growing (along with their tumor), progressively lose motility and ultimately die [11]. 

The developmental arrest of *Ras^V12^; scrib^−/−^* tumor-bearing larvae was the first evidence that these tumors have systemic effects and impair the developmental progression and physiology of the whole organism. 

*Drosophila* develops through three successive larval stages (namely L1, L2, and L3) before initiating pupation, during which metamorphosis reshapes the larval structures into finalized adult organs. Molts, pupation, and metamorphosis are tightly controlled by regulated bursts of production of the steroid hormone Ecdysone by the prothoracic gland [119]. Ecdysone production is finely balanced by systemic growth inputs which ensure proper organism size and symmetry control. However, upon damage of larval structures, Ecdysone production (and consequently development) is put on hold while systemic growth is slowed down until the wound is repaired. The insulin-like peptide Dilp8 is the main messenger that triggers developmental arrest upon tissue damage. For instance, damaged imaginal discs overexpress and secrete Dilp8 in the hemolymph [120,121]. Dilp8 binds to the relaxin receptor Lgr3 (Leucine-rich repeat-containing G-protein-coupled receptor 3) in the GCL neurons in the brain. These neurons activate a circuit that ultimately abrogates Ecdysone production in the prothoracic gland [122,123,124,125]. 

An old concept in cancer research is that tumors can be considered as wounds that do not heal. In line with this idea, *Ras^V12^; scrib^−/−^* tumor-bearing eye discs (and other imaginal disc tumor models) overexpress and secrete Dilp8 [72,121,126]. As a result, Ecdysone production decreases, and pupation is blocked. How Dilp8 expression is induced by *Ras^V12^; scrib^−/−^* eye discs tumors is not entirely clear. Interestingly, the JNK and SWH pathways are the primary potential regulators of Dilp8 expression. Indeed, Dilp8 overexpression in *Ras^V12^; scrib^−/−^* eye disc tumors is strongly down-regulated upon loss of function of the main JNK effectors *fos*, *ets21c*, and *ftz-f1* and is accompanied by a rescue of both the pupation defect and adult survival [72]. 

Additionally, it has recently been shown that elevated *Ras^V12^* in the eye discs leads to increased JNK signaling and further dilp8 upregulation in late larval stages. Although not able to prevent pupation alone, *Ras^V12^*-mediated Dilp8 elevation lowers Ecdysone production so that after pupation, metamorphosis cannot proceed and the pupae die [49]. It is thus tempting to speculate that in *Ras^V12^; scrib^−/−^* eye disc tumors JNK signaling would be a major regulator of dilp8 expression and Ecdysone signaling impairment. However, a study in the wing disc strongly supports the idea that although responsive to JNK signaling, *dilp8* transcriptional activity is mainly controlled by the Yki partner Sd, which binds to specific sites in its enhancer region [127]. Given the crucial role of Yki for *Ras^V12^; scrib^−/−^* tumor proliferation and invasion, future work might also uncover its contribution to the pupation defects together with JNK. 

Nevertheless, JNK signaling inhibition in *Ras^V12^; scrib^−/−^* tumors restores pupation and improves adult survival, demonstrating the involvement of this pathway in the developmental delay phenotype [72]. Noteworthy, this phenomenon might also occur in a Dilp8-independent manner. Like, Dilp8, Upd3 has recently been implicated in inducing a developmental delay in a chromosomal instability (CIN)—driven wing disc tumor model [128]. In this model, secreted Upd3 acts as a systemic signal that promotes a developmental delay through JAK-STAT signaling and bantam microRNA upregulation in the prothoracic gland. Consequently, Ecdysone biosynthesis decreases, and the metamorphosis onset is delayed [128]. Because upd3 is strongly upregulated in *Ras^V12^; scrib^−/−^* tumors in a JNK-dependent manner, a similar Ecdysone-inhibiting mechanism might occur due to Upd3 expression and contribute to the lingering phenotype [86].

### 5.2. Innate Immune Response: The Tumor as a Wound That Never Heals

*Ras^V12^; scrib^−/−^* not only triggers damage-like development arrest, it also instigates a strong systemic inflammation response.

#### 5.2.1. The Enigmatic Role of the Tumor-Associated Hemocytes

In *Drosophila*, the immune response is partially mediated by the macrophage-like cells called hemocytes. They are a primary line of defense against fungi, bacteria, and yeast. The three types of hemocytes: the plasmatocytes, the lamellocytes, and the crystal cells, execute phagocytosis/coagulation, encapsulation, and melanization, respectively [129]. Upon infection, hemocytes activate the fat body’s systemic immune response through the cytokine Upd3. The fat body (Figure 1A) (whose function is similar to the mammalian liver) activates the Toll or IMD NFkB pathways and massively secrete pathogen-tailored anti-microbial peptides into the hemolymph [129].

In *Ras^V12^; scrib^−/−^* tumor-bearing larvae (as well as in *scrib^−/−^* and *dlg^−/−^* larvae which display neoplastic growth in the imaginal discs), the number of circulating hemocytes increases due to higher hemocyte proliferation. Plasmatocytes adhere to the surface of *Ras^V12^; scrib^−/−^* tumors where the basement membrane is degraded, similarly to injured disc, emphasizing the notion that these tumors are perceived as wounds by the organism [7,8,70,74,77,130]. Investigating the role of these tumor-associated hemocytes (TAH) is technically and genetically challenging. Several studies involving demanding techniques such as tumor transplantation or hemocytes transfusion have elegantly addressed this question. Unfortunately, they do not reach the same conclusions and report both a pro and an anti-tumorigenic role of the *Ras^V12^; scrib^−/−^* TAHs.

To follow tumor growth over a long time inside a host (instead of 15 days in the larva), a common technique consists of cutting and transplanting tumor pieces into the abdomen of an adult female fly [131]. When transplanted, *Ras^V12^; scrib^KD^* wing disc tumors attract adult hemocytes which restrict tumor growth [77]. Similarly, neoplastic tumors from whole *scrib^−/−^* eye discs as well as *dlg^−/−^* eye discs (which display neoplastic growth) grow better when the TAHs are killed in the larva [8,130]. Interestingly, the TAHs associated with eye disc tumors in *dlg^−/−^* mutant larvae, secrete both Egr and Spz. In this context, Egr triggers local tumor cell apoptosis suggesting an anti-tumorigenic role of TAHs in *scrib^−/−^* and *dlg^−/−^* mutant larvae [8,130,132]. By contrast, in *Ras^V12^; scrib^−/−^* eye disc tumor-bearing larva, the TAHs also secrete Eiger and contribute to JNK signaling through MMP1 expression within the tumor arguing for a tumor-invasion supportive role [7] although no observation on tumor growth has been made. JNK (Cher) and JAK-STAT (STAT-GFP) signaling have been observed in TAHs but not further investigated [8,70]. These opposite potential roles of TAHs might be inherent to the type of tumors they associate with. Indeed, JNK signaling is protumorigenic only when *Ras^V12^* is expressed and inhibits apoptosis. It is, therefore, tempting to speculate that in *Ras^V12^; scrib^−/−^* tumors, TAHs might be tumorigenic through the promotion of JNK signaling in the tumor, whereas TAHs are antitumorigenic in *scrib^−/−^* and *dlg^−/−^* larvae for the very same reason. Future investigations would be required to elucidate these questions.

A related question is how TAHs are recruited to the tumor. *Ras^V12^; scrib^−/−^* tumors produce ROS in a JNK-dependent manner. ROS are particularly enriched where the basement membrane is degraded through the activity of the transmembrane NADPH oxidase Duox [74]. In *Ras^V12^; scrib^−/−^* tumors, ROS production inhibition both intracellularly and extracellularly abrogates TAHs recruitment. Interestingly, a similar hemocyte recruitment mechanism is observed in the eye discs bearing undead cell clones suggesting that the *Ras^V12^, scrib^−/−^* tumor growth relies partly on AiP mechanisms and that hemocytes are part of this process [133].

#### 5.2.2. NFκB Activation in the Tumor Triggers a Humoral Inflammation Response

Another part of the inflammation response is characterized by the NFκB-mediated elevation of AMPs in the hemolymph. TAHs in *dlg^−/−^* mutant larvae triggers a long range Toll-mediated inflammation response in the fat body by secreting Spz [130]. Indeed, Spz activates Toll signaling in the fat body and trachea which results in the secretion of the anti-microbial peptide (AMP) Defensin. Interestingly, secreted Defensin attach to phospatidylserines that are exposed at the surface of the tumor in an Eiger-dependent manner ultimately provoking tumor cell death [132]. Moreover, a recent study has shown that *Ras^V12^; scrib^−/−^* tumor-bearing larvae have elevated levels of the AMP Drosomycin, also a target of the Toll pathway, confirming that the presence of the tumor within the larva induces a systemic inflammation response [134]. Interestingly, bacterial but not fungal infection of *Ras^V12^; scrib^−/−^* tumor-bearing larvae can modulate this inflammation and results in weaker tumor growth, suggesting that inflammation restricts tumor growth [134]. Recently, an elegant RNAi-mediated knock-down screen has been performed *in vitro* on *Ras^V12^; scrib^−/−^* cells challenged with a scratch assay to characterize novel invasion regulators. Toll6, one of the receptors of the Toll pathway, is enriched in the tumor and governs the trajectory of invasive *Ras^V12^; scrib^−/−^* cells toward specific organs (aka organotropism), through its binding to the ligand Spz. Strikingly, only the Spz-expressing larval organs are susceptible to secondary tumor formation [53] suggesting that NFκB signaling has an unexpected role in cancer invasion. Finally, a recent study reported that the negative NFκB regulator Cactus is also enriched in *Ras^V12^; scrib^KD^* wing disc tumors. In this context, Cactus is proposed to contribute to tumor growth independently of the NFκBs but rather through the promotion of Yki signaling. Yet, additional studies would be required to validate and assess the relevance of this unconventional mechanism [94]. 

To conclude, a cohort of studies report that *Ras^V12^; scrib^−/−^* tumors initiate an inflammation response within the larvae with an elevation of specific AMPs and the potential involvement of components of the NFκB pathways. This is thought to modulate tumor growth and invasion. Additionally, hemocytes are attracted to the tumor, and the nature of this crosstalk involves ROS production and Eiger secretion [7,74]. However, the function of this association still needs to be clarified in the different tumor models.

Nevertheless, these observations emphasize that the *Ras^V12^; scrib^−/−^* tumor model is also suited for studying the role of innate immunity in cancer. Given the high degree of conservation of the NFκB pathway, future findings might contribute to understanding the role of innate immunity in human tumorigenesis.

### 5.3. Wasting through Systemic Induced Autophagy Is Needed for Tumor Growth

*Ras^V12^; scrib^−/−^* tumors can grow in adult fly hosts upon allograft transplantation and eventually kill the host. *Ras^V12^; scrib^−/−^* tumors lead to abdominal bloating, shrinkage of the fat body, altered mitochondrial morphology of muscles, and ovary wasting [11,126]. These changes are reminiscent of systemic effects during cancer cachexia in humans.

The functional relevance of this phenotype is still unclear; however, it seems to be a shared characteristic among several neoplastic tumors [135,136].

*Ras^V12^; scrib^−/−^* tumor-bearing larvae suffer weight loss, impaired motility and decreased feeding over time [115,137]. The energy-storing organs, such as the muscle and the fat body in the larva, progressively deteriorate over time so that many muscles shrink or rupture, and adhesion of fat body cells decreases and attains a “pearl-like” phenotype during the late stages of tumorigenesis [115,126,137]. The reduction of energy storage accompanies structural defects in these organs. For instance, glycogen storage is significantly reduced in the muscle and the fat body and accompanied by mitochondrial defects. In contrast, lipid storage as triacylglycerides in the fat body seems to be stable, as lipid-free cytoplasm shrinks and lipid droplet aggregation ensues [115,126]. These changes correlate with an increase of amino acid and sugar levels in the larval hemolymph (insect blood), a phenotype shared with a high-sugar diet-induced *Ras^V12^, csk^−/−^* larval tumor model [115,135,136,137,138]. The hyperglycemia and amino acid elevation in the hemolymph appear before the onset of alterations in feeding. This observation suggests that nutrient mobilization from wasted organs in the *Ras^V12^; scrib^−/−^* tumor-bearing larvae is mainly attributed to a long-range action of the tumor rather than starvation [115,137]. Collectively, this cohort of alterations of the organ structure and the systemic metabolism defines a wasting phenotype that is also observed in cancer patients who suffer from cachexia, establishing *Drosophila* as a relevant model to investigate the cachexia-associated mechanism (reviewed in [139]).

This wasting phenotype raises two main questions. (1) How does the tumor induce organ wasting? (2) Does organ wasting contribute to tumor growth?

#### 5.3.1. Tumors Secrete Wasting-Inducing Ligands

Initial studies aimed to answer the first question. Transplanted *Ras^V12^; scrib^−/−^* tumors secrete the insulin growth factor binding protein (IGFBP) homolog ImpL2 which mediates organ wasting [126]. The mechanism through which tumor-secreted ImpL2 mediates ovary wasting was further detailed using an additional Yki-induced adult gut model [136]. In both models, ImpL2 executes muscle wasting by decreasing systemic Insulin Receptor-PI3K-AKT signaling, and downregulating energy production genes involved in glycolysis, pyruvate metabolism and oxidative phosphorylation in the muscle. Interestingly, the ImpL2 regulatory region possesses binding sites for both Fos and Scalloped suggesting that in *Ras^V12^; scrib^−/−^* eye disc tumors both the JNK and the SWH pathways might control its expression [126]. However, ImpL2 is not the sole mediator of wasting in *Drosophila*. In the Yki-induced gut tumor model, tumors also secrete the Pvf1 (PDGF- and VEGF-related factor 1) which is thought to trigger adipose and muscle wasting through Pvr/ERK signaling [135]. In the *Ras^V12^, csk^−/−^* high-sugar diet-induced tumor model, systemic muscle wasting is neither governed by ImpL2 nor by Pvf1. Instead, the fibroblast growth factor Branchless (Bnl) is secreted by the tumor and mediates both muscle wasting and tumor growth [138]. Thus, it would be interesting to decipher whether Pvf1 or Bnl also contribute to the wasting phenotype induced by *Ras^V12^; scrib^−/−^* tumors. 

**Figure 4:**
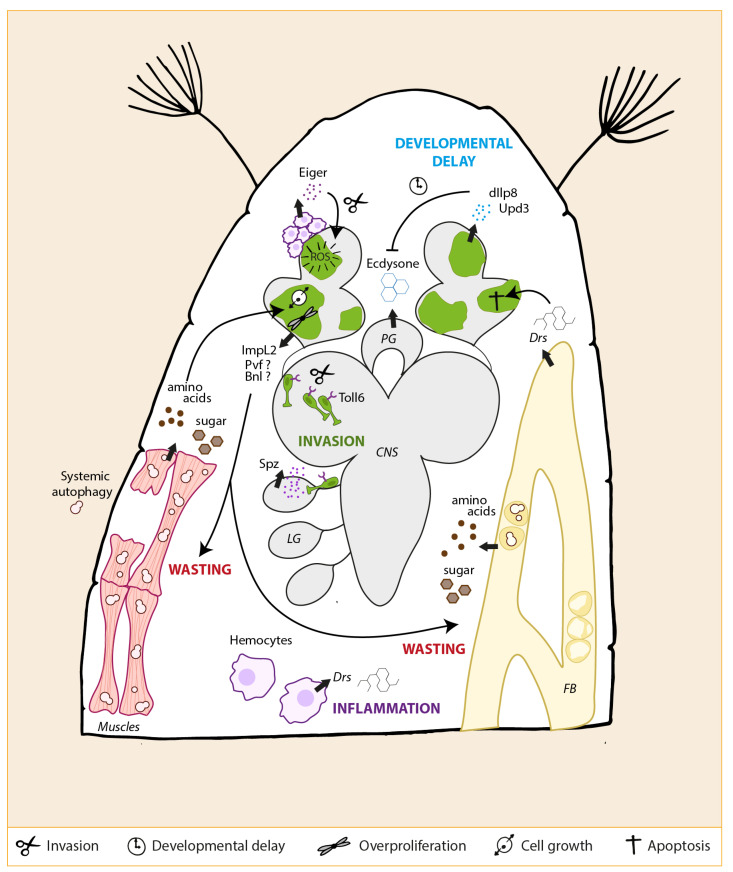
*Ras^V12^; scrib^−/−^* eye antennal disc tumors alter distant organ functions. Invasion: *Ras^V12^; scrib^−/−^* tumors (green) invade adjacent organs such as the central nervous system (CNS) and the leg discs (LD). Invasion is guided by the interaction between the Toll6 receptor at the surface of invasive tumor cells and its ligand Spz, secreted by a subset of organs prone to secondary tumor formation. Developmental delay: It relies on the decrease of Ecdysone production by the prothoracic gland (PG). The tumor cells secrete Dilp8 and Upd3, which are both known for their ability to inhibit Ecdysone production. Inflammation: Tumor cells (green) have elevated ROS levels, attracting plasmatocytes (purple) to the basement membrane breach. These plasmatocytes secrete Eiger and contribute to mmp1 expression in the tumor cells and subsequently to their ability to escape the primary tumor, arguing for a pro tumorigenic function. Together with the fat body, they constitute the potential source of the AMP Drosomycin that is found elevated in the larval hemolymph. Drosomycin (Drs) triggers cell death in the tumor, arguing for a potential anti-tumorigenic role of the plasmatocytes. Wasting: Tumor cells secrete ImpL2 (and potentially Pvf and Bnl), which is thought to trigger a wasting phenotype in the larva characterized by the morphological deterioration of the energy-storing organs: the muscles (shrinkage and split) and the fat body (pearl-like phenotype, lipid droplet aggregation). This is accompanied by elevation of autophagy in these organs as well as elevated sugar and amino acid levels in the hemolymph. In its late stage, the tumor building blocks derive from host to maintain its growth.

In conclusion, it appears that depending on the tumor type, various systemic signals are generated by the transformed cells in order to orchestrate a common host metabolic reprogramming which results in the progressive wasting of energy storage organs (Figure 4). The exact nature of the crosstalk between the tumor and the targeted organs is still an open question.

#### 5.3.2. Organ Wasting Feeds the Tumor

A long-standing question has been whether tumor-associated organ wasting is an unintended side effect, or whether it serves a specific purpose for tumor growth, be it through cell signaling or nutrient supply. A recently described stable carbon isotope tracing technique has allowed to distinguish whether tumor biomass is derived from food or host tissues. Using this technique, it was demonstrated that *Ras^V12^; scrib^−/−^* larval tumors first grow mainly on nutrients from food and progressively incorporates more nutrients from host tissues [115,140]. Autophagy is aberrantly activated in muscles and fat body of *Ras^V12^; scrib^−/−^* tumor-bearing larvae suggesting that wasted organs might provide nutrients to the tumor through “organ self-digestion” [54]. Indeed, systemic autophagy inhibition demonstrated that autophagy is required for organ wasting and elevated amino acid and sugar levels in hemolymph that supports tumor growth [115]. Thus, *Ras^V12^; scrib^−/−^* tumors consume nutrients originating from wasting organs, although the exact source of nutrients and how tumors activate systemic autophagy and organ wasting is still unclear.

## 6. Conclusions

The cooperation between the oncogene *Ras^V12^* and the loss of the tumor suppressor *scribble* gives rise to invasive malignant tumors in the *Drosophila* eye-antennal disc. In this model, tumor cells adopt a disorganized and altered morphology accompanied with the aberrant activation of key signaling pathways (JNK, Yki, Raf/Mek/MAPK, PI3K/AKT and JAK-STAT). This cocktail of mitogenic signals fuels the neoplastic tumor growth which in turn severely alters the tissue integrity through dismantling cell adhesion, degrading the basement membrane, counteracting differentiation as well as stimulating cell growth and ectopic proliferation. Finally, the tumor alters the whole-body physiology of the host by impairing hormonal signaling, halting host development, invading and damaging local and distant organs, triggering a systemic inflammation response and consuming the nutrients from the degradation of host tissues. Together, this results in loss of body motility, decrease in feeding and ultimately death of the host.

Unexpectedly, this originally simple *in vivo Drosophila* tumor model, which has been elaborated upon for nearly two decades, has brought insights into tumorigenesis that involves conserved components, associated with malignant cancer progression and bad prognosis in human cancer (loss of cell polarity, aberrant activation of conserved signaling pathways, ROS production, NFκB inflammatory signaling and organ wasting). Excitingly, the main pathways involved in *Ras^V12^; scrib^−/−^* tumorigenesis also turn out to be a common feature of most of the other *Drosophila* cooperative oncogenic models [141], suggesting that these conserved signaling pathways might also be a common conserved underlying transformation program critical for driving genetic cooperation in human cancers. 

Still, many unexplored questions need to be addressed, in particular the nature and functions of the distinct communication signals between the tumor cells and their immediate wild type neighboring cells or distant wasting organs. How does tumors outcompete and possibly consume neighboring cells by supercompetition? What are the mechanisms of metabolic reprogramming of tumor and host? How do tumor cells inflict harm on the host and lead to death? Effectively addressing these and other questions of tumor–host dialogue will require combinatorial genetic manipulation techniques that will allow both the generation of *Ras^v12^*; *scrib^−/−^* tumors (or other tumor models) and the genetic manipulation of any cellular process in selected organs *in vivo*.

We predict that the variety of synergistic tumor models combined with novel genetic approaches available in *Drosophila*, coupled with increasingly sensitive omic approaches will keep on contributing to the acquisition of precious novel and transferrable knowledge and concepts in cancer biology.
